# Individual and Combined Cytotoxic Effects of Zearalenone and Zearalenone-14-Glucoside in Porcine Intestinal Epithelial Cells

**DOI:** 10.3390/vetsci13090885

**Published:** 2026-08-30

**Authors:** Rui Liu, Fuqing Liu, Wenjing Liu, Tao Li, Jiayuan Xie, Chunxi Fu, Xuejun Yuan, Weiren Yang, Ning Jiao

**Affiliations:** 1 Key Laboratory of Efficient Utilization of Non-Grain Feed Resources (Co-Construction by Ministry and Province), Ministry of Agriculture and Rural Affairs, College of Animal Science and Technology, Shandong Agricultural University, Tai’an 271017, China; 2 Animal Husbandry and Veterinary Workstation in Daotian Town, Shouguang 262728, China; 3 College of Life Sciences, Shandong Agricultural University, Tai’an 271018, China

**Keywords:** zearalenone, zearalenone-14-glucoside, combined toxicity, intestinal porcine epithelial cell-jejunal 2, cell viability

## Abstract

Zearalenone (ZEN) and its masked form, zearalenone-14-glucoside (Z14G), are common mycotoxins found in feed that can threaten the intestinal health of piglets. To date, there has been a lack of systematic research on the toxic effects and molecular mechanisms of Z14G alone and in combination with ZEN on intestinal porcine epithelial cell-jejunal 2 (IPEC-J2) cells. This study found 80 µmol/L ZEN and 100 µmol/L Z14G were the selected experimental concentrations based on cell viability assays. Both ZEN and Z14G significantly inhibited cell viability and proliferation, induced apoptosis, and disrupted the expression of associated genes. They altered the expression of barrier-associated genes, evidenced by increase in lactate dehydrogenase release and downregulation of tight junction gene mRNA. ZEN and Z14G exposure increased oxidative stress, altered antioxidant defense, and modulated inflammatory responses, accompanied by changes in the expression of genes related to the TLR4/MyD88 and NLRP3 signaling pathways. Notably, Z14G alone exhibited a distinct transcriptional response of inflammatory-related genes, failing to upregulate *IL-1β*, *TNF-α* and *STING*. In conclusion, ZEN and Z14G disrupt intestinal epithelium homeostasis via interlinked oxidative, inflammatory and barrier-damaging pathways. The findings reveal the direct toxicity and unique properties of ZEN and Z14G, underscoring the need to consider masked mycotoxins in combined risk assessment.

## Simple Summary

Zearalenone (ZEN) is a common mycotoxin in animal feed that can harm livestock health. This study investigated the toxic effects of ZEN and its “masked” form zearalenone-14-glucoside (Z14G) on enterocytes of pigs. We found that the damage to porcine intestinal epithelial cells caused by combined exposure of ZEN and Z14G was significantly more serious than that caused by exposure to either one alone. More importantly, our findings demonstrate that Z14G alone can exert cytotoxic effects on porcine intestinal epithelial cells, even in the absence of ZEN. This discovery illustrates the toxic effects of ZEN and its masked form, providing a new theoretical basis for more accurate risk assessment of mycotoxins to ensure feed safety.

## 1. Introduction

Mycotoxin contamination in feedstuff poses a persistent threat to global animal husbandry and food safety [1]. Zearalenone (ZEN), a potent estrogenic mycotoxin produced by Fusarium species, is frequently detected in cereal-based feeds [2]. Upon ingestion, ZEN can be metabolized into various modified forms [3,4]. Notably, zearalenone-14-glucoside (Z14G) is a major masked mycotoxin formed in plants as a conjugate. This modification was once considered a detoxification pathway in plants [5,6]. However, emerging evidence indicates that Z14G can be hydrolyzed back to ZEN in the digestive tract, potentially acting as a hidden source of toxicity and leading to underestimated risk [7,8].

The intestine, as the primary site of ZEN absorption, is also ZEN foremost toxicological target. Short-term ZEN exposure disrupted the gut microbiota, impaired mucosal immunity, induced local inflammation and caused structural damage [9]. At the cellular level, ZEN triggered oxidative stress and reduced the expression of tight junction proteins (e.g., ZO-1) in a dose-dependent manner, which was associated with cytoskeletal rearrangement via RhoA/ROCK pathway activation [9,10]. Beyond damaging the physical barrier, ZEN exacerbated immune toxicity, such as by promoting myeloid-derived cell proliferation via the 27-HC/LXRs/ApoE axis, thereby inhibiting T-cell activity [11]. Furthermore, ZEN-induced dysbiosis, characterized by a reduction in butyrate-producing bacteria, contributed to systemic inflammation [12]. Although low direct toxicity, Z14G could be rapidly hydrolyzed to bioactive ZEN (and its reduced metabolites, α/β-ZEL) by various mechanisms, including intestinal β-glucosidases, hepatic carboxylesterases, and serum albumin, then significantly increased overall exposure [13,14,15]. Notably, emerging evidence suggested that Z14G may also possess intrinsic toxicity, as high concentrations could reduce cell viability and cause pathological gut-associated lymphoid tissue hyperplasia, although its effects appeared dose- and cell type-dependent [7,13,16]. Despite this, the direct intestinal toxicity of Z14G, especially its combined effects with ZEN, remains poorly understood, creating a critical gap in the risk assessment.

Therefore, this study aimed to investigate and compare the individual and combined toxic effects of ZEN and Z14G on intestinal porcine epithelial cell-jejunal 2 (IPEC-J2). The findings provide fundamental insights into the combined toxicity mechanisms of ZEN and its masked form and offer a theoretical basis for a more scientific risk assessment of mycotoxin contamination.

## 2. Materials and Methods

Zearalenone (ZEN) and Zearalenone-14-glucosid (Z14G) were purchased from Shanghai Zhenzhun Biotechnology Co., Ltd. (Shanghai, China). The intestinal porcine epithelial cells (IPEC-J2) were purchased from BeNa Culture Collection (Beijing, China).

### 2.1. Cell Culture and Treatments

The IPEC-J2 cells were cultured in Dulbecco’s modified Eagle’s medium (DMEM, Gibco, Waltham, MA, USA) containing 10% fetal bovine serum (FBS, Excell Bio, Montevideo, Uruguay) and 1% streptomycin-penicillin (PS, Gibco, Waltham, MA, USA), and cultured at 37 °C in 5% CO_2_. The cell culture solution was changed every 1–2 days. Cell passaging and freezing were performed when cell fusion reached 80% of the dish. The cells were seeded in 96-well plates and allowed to reach 70% confluence for subsequent analysis. ZEN and Z14G were dissolved in dimethyl sulfoxide (DMSO, Amresco, Solon, OH, USA) as a stock solution and stored at −20 °C.

### 2.2. Assay of Cell Viability

IPEC-J2 cells were seeded in 96-well plates in DMEM at a density of 1 × 10^4^ cells/well. When the cells reached approximately 70% confluence, they were starved for 6 h and then treated with ZEN (0, 40, 80, 100, 150, or 200 μmol/L) or Z14G (0, 40, 80, 100, 150, or 200 μmol/L) for 24 h. Cell viability was assessed to characterize the cytotoxic effects of ZEN and Z14G and to select concentrations for subsequent experiments. The treatment duration was selected based on a previous study [17]. After treatment, 10 μL of CCK-8 reagent (Beyotime, Shanghai, China) was added to each well under light-protected conditions, followed by incubation at 37 °C for 2 h. The optical density (OD) at 450 nm was measured using a microplate reader, and cell viability was calculated accordingly. To exclude potential direct interference of ZEN or Z14G with the CCK-8 assay chemistry or optical readout, cell-free controls containing the corresponding concentrations of ZEN or Z14G and CCK-8 reagent were included and processed under the same conditions. No cells were added to these control wells. Based on the dose–response results and the suitability of the observed cytotoxic effects for subsequent mechanistic analyses, 80 μmol/L ZEN and 100 μmol/L Z14G were selected for the subsequent experiments. These concentrations were selected as experimental concentrations and were not intended to represent equivalent biological doses.

### 2.3. Assay of Cell Proliferation

The IPEC-J2 cells were seeded in 96-well plates (1 × 10^4^ cells/well) and allowed to reach 70% confluence, and were then starved for 6 h. Cells in each cytotoxic group were treated with ZEN and Z14G at the selected experimental concentrations for 24 h. Subsequently, the cell proliferation was measured using the EdU cell proliferation assay kit (RiboBio, Guangzhou, China) according to the manufacturer’s instructions. The proliferating cells were stained red with Apollo, whereas the cell nuclei were stained blue with Hoechst 33342. Finally, the plate was observed under a fluorescence microscope (RIDET, Shanghai, China). The proliferating cells and cell nuclei were counted separately and the red to blue ratio was calculated to determine the proliferation rate of cells.

### 2.4. Measurement of Cell Apoptosis

The IPEC-J2 cells were seeded in 6-well plates at a density of 1 × 10^5^ cells/well and allowed to reach approximately 70% confluence before treatment with ZEN and Z14G for 24 h. Subsequently, viable, apoptotic, and necrotic cell populations were quantified using an Annexin V-FITC Cell Apoptosis Assay Kit (Beyotime, Shanghai, China) according to the manufacturer’s instructions. Briefly, the cells were collected and co-stained with Annexin V-FITC and propidium iodide (PI), followed by flow cytometric analysis (Becton, Dickinson and Company, Franklin Lakes, NJ, USA). Based on the differential staining patterns, four cell populations were identified: Annexin V-FITC−/PI− cells as viable cells, Annexin V-FITC+/PI− cells as early apoptotic cells, Annexin V-FITC+/PI+ cells as late apoptotic/secondary necrotic cells, and Annexin V-FITC−/PI+ cells as necrotic cells. The total apoptotic cell population was calculated as the sum of early apoptotic and late apoptotic/secondary necrotic cells. Flow cytometric data were analyzed using FlowJo V10 (Tristar, Brea, CA, USA).

### 2.5. Assessment of Intracellular Oxidative Stress

The IPEC-J2 cells were seeded in 96-well plates at a density of 1 × 10^4^ cells/well and allowed to reach approximately 70% confluence before exposure to ZEN and Z14G for 24 h. Intracellular oxidative stress was assessed using a ROS assay kit (Beyotime, Shanghai, China) according to the manufacturer’s instructions. After treatment, the cells were incubated with 10 μmol/L DCFH-DA at 37 °C for 20 min in the dark and then washed three times with serum-free medium. Fluorescence images were captured using a fluorescence microscope (RIDET, Shanghai, China), and the fluorescence intensity was quantified using ImageJ software (version 1.54p, NIH, Bethesda, MD, USA).

### 2.6. LDH Assay

Cell seeding was performed as described in Section 2.4. After drug exposure, the culture medium was collected and used for the determination of extracellular lactate dehydrogenase (LDH) activity. LDH activity in the culture supernatant was measured using an LDH assay kit (A020-2, Nanjing Jiancheng Bioengineering Institute, Nanjing, China) according to the manufacturer’s instructions. The absorbance was measured at 450 nm using a microplate reader, and LDH activity was calculated according to the manufacturer’s formula and expressed as U/L. The untreated cells were used as the negative control. No separate maximum-release control was included in the original experimental design; therefore, LDH activity was reported as absolute extracellular LDH activity (U/L) rather than as a percentage of maximum LDH release.

### 2.7. Determination of Antioxidant Parameters

IPEC-J2 cells were seeded in 100 mm plates in DMEM and allowed to reach 70% confluence, and then treated for 24 h according to the above treatments. After treatments, the cells were digested with trypsin, and the cell precipitate was prepared in pre-cooled saline. The cells were then broken using a freezing homogenizer (SCIENTZ, Ningbo, China), followed by centrifugation at 3000 r/min for 10 min. The supernatant was collected and analyzed for T-AOC and CAT levels using respective assay kits (Jiancheng Bioengineering Co., Nanjing, China) according to the manufacturer’s instructions.

### 2.8. Reverse Transcription PCR and Quantitative Real-Time PCR

IPEC-J2 cells were seeded in 100 mm plates in DMEM and allowed to reach approximately 70% confluence before treatment according to the experimental conditions described above. After treatment, cells were collected, and total RNA was extracted using AIPzol total RNA extraction reagent (i-presci, Shanghai, China) according to the manufacturer’s instructions. RNA concentration and purity were assessed using an RNA concentration tester, and RNA integrity was evaluated by agarose gel electrophoresis using the GelDoc Go Gel Imaging System (Bio-Rad, Hercules, CA, USA). RNA samples with acceptable A260/A280 and A260/A230 ratios and intact RNA bands were used for subsequent analysis. Genomic DNA was removed and cDNA was synthesized using the Evo M-MLV Reverse Transcription Reagent (Accurate Biology, Suzhou, China). Quantitative real-time PCR was performed using a QuantStudio™ 5 Real-Time PCR System (Thermo Fisher Scientific, Waltham, MA, USA) with SYBR Green Pro Taq HS Premixed qPCR Reagent (Accurate Biology, Suzhou, China). Primer amplification efficiency was evaluated using serial dilutions of cDNA, with efficiencies ranging from 93.5% to 105.8% and R^2^ values ranging from 0.992 to 0.999. Melt-curve analysis showed a single specific peak for each primer pair. No-template controls were included in each qPCR run, and no detectable amplification was observed. β-actin was used as the reference gene based on its stable expression across the experimental groups. Relative mRNA expression levels were calculated using the 2^−ΔΔCt^ method. The primer sequences are listed in Table 1.

### 2.9. Statistical Analysis

Statistical analysis of data was carried out using one-way analysis of variance (ANOVA) in SAS 9.4 (SAS Institute Inc., Cary, NC, USA). The multiple comparison test among the treatments was performed by Tukey’s method. The figures were drawn by GraphPad Prism 9.0 (GraphPad Prism Software Inc., Boston, MA, USA). For each assay, *n* = 6 represents six independent biological replicates. Where technical replicates were performed, measurements were averaged within each biological replicate before statistical analysis. The results were presented as means ± SEMs. All statements of significance were based on a probability of a *p*-value < 0.05.

## 3. Results

### 3.1. Effects of ZEN and Z14G on Cellular Homeostasis and Renewal in IPEC-J2 Cells

#### 3.1.1. Cell Viability and Proliferation

**Figure 1:**
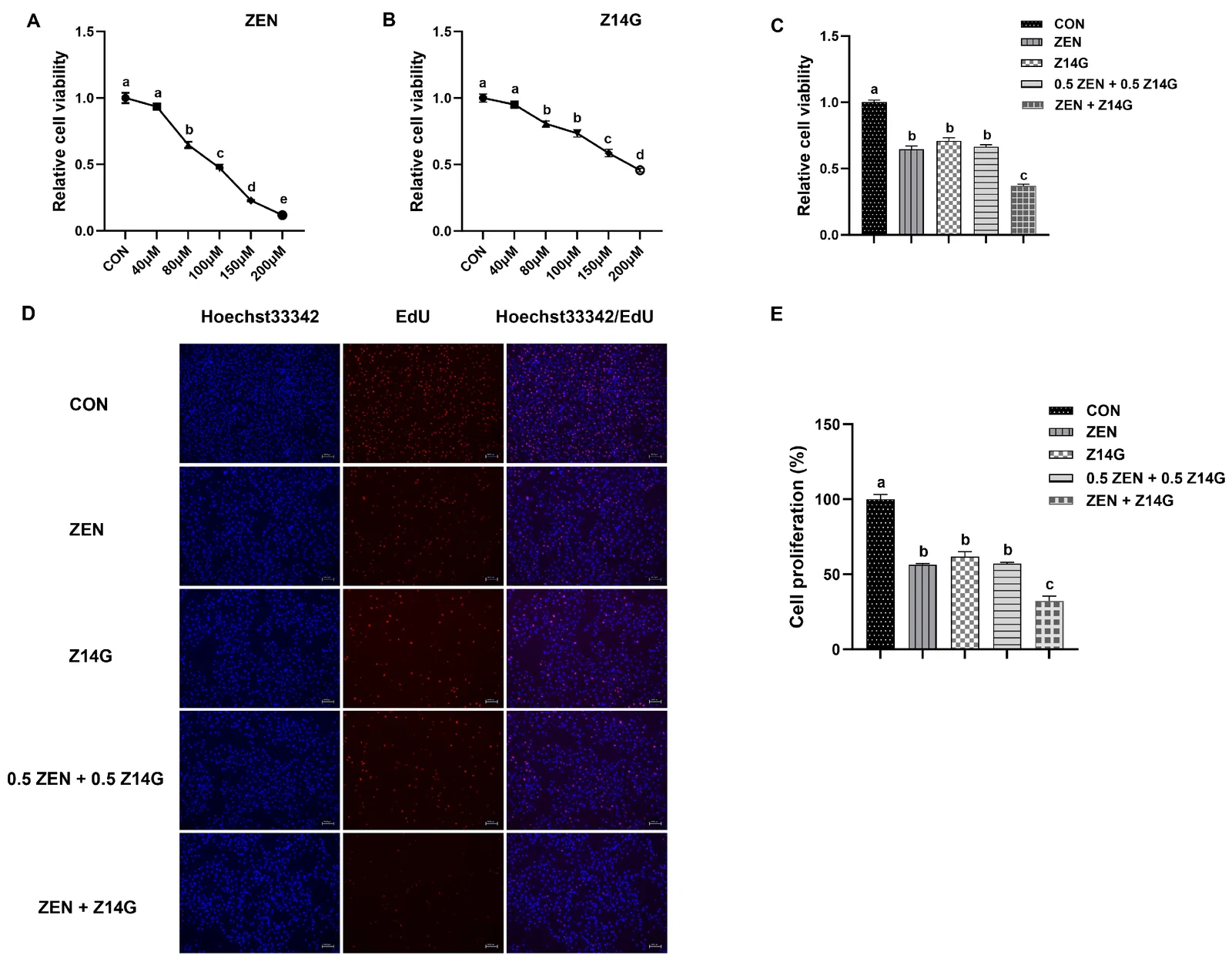
Effects of ZEN and Z14G exposure on cell viability and proliferation. (**A**) represents the effects of ZEN gradient concentration exposure on cell viability. (**B**) represents the effects of Z14G gradient concentration exposure on cell viability. (**C**) represents the effects of individual and combined ZEN and Z14G exposure on cell viability. (**D**) represents the EdU assay for cell proliferation. (**E**) represents the quantification of (**D**). Hoechst 33342-labeled nuclei are shown in blue and EdU-labeled proliferating cells are shown in red. *n* = 6. ^a–e^ Different lowercase letters indicate significant differences (*p* < 0.05).

The effects of ZEN and Z14G alone and in combination on IPEC-J2 cell viability and proliferation are shown in Figure 1. Cell viability of IPEC-J2 cells is shown in Figure 1A–C. Compared with the CON group, ZEN exposure significantly reduced cell viability in a dose-dependent manner, with reductions of 7%, 36%, 53%, 78% and 88% at concentrations of 40, 80, 100, 150 and 200 μmol/L, respectively. In contrast, Z14G exhibited a milder cytotoxic effect, decreasing cell viability by 5%, 20%, 30%, 42% and 55% at concentrations of 40, 80, 100, 150 and 200 μmol/L, respectively. Doses of 80 μmol/L ZEN and 100 μmol/L Z14G were confirmed for the following study. Furthermore, cell viability was significantly reduced by 34% and 64% by the combination of 40 μmol/L ZEN with 50 μmol/L Z14G (0.5 ZEN and 0.5 Z14G) and 80 μmol/L ZEN with 100 μmol/L Z14G (ZEN and Z14G), respectively (*p* < 0.05). The cell-free toxin + CCK-8 controls showed no appreciable interference with the CCK-8 reaction or the OD450 nm readout at the concentrations tested. The proliferation of IPEC-J2 cells is shown in Figure 1D,E. Compared with the CON group, treatment with ZEN, Z14G, 0.5 ZEN + 0.5 Z14G, or ZEN + Z14G significantly reduced the number of EdU-positive cells and inhibited the proliferation of IPEC-J2 cells (*p* < 0.05). Among all treatments, the ZEN + Z14G group exhibited the most pronounced inhibitory effect on cell proliferation, as evidenced by the lowest number of EdU-positive cells (*p* < 0.05).

#### 3.1.2. Cell Apoptosis

**Figure 2:**
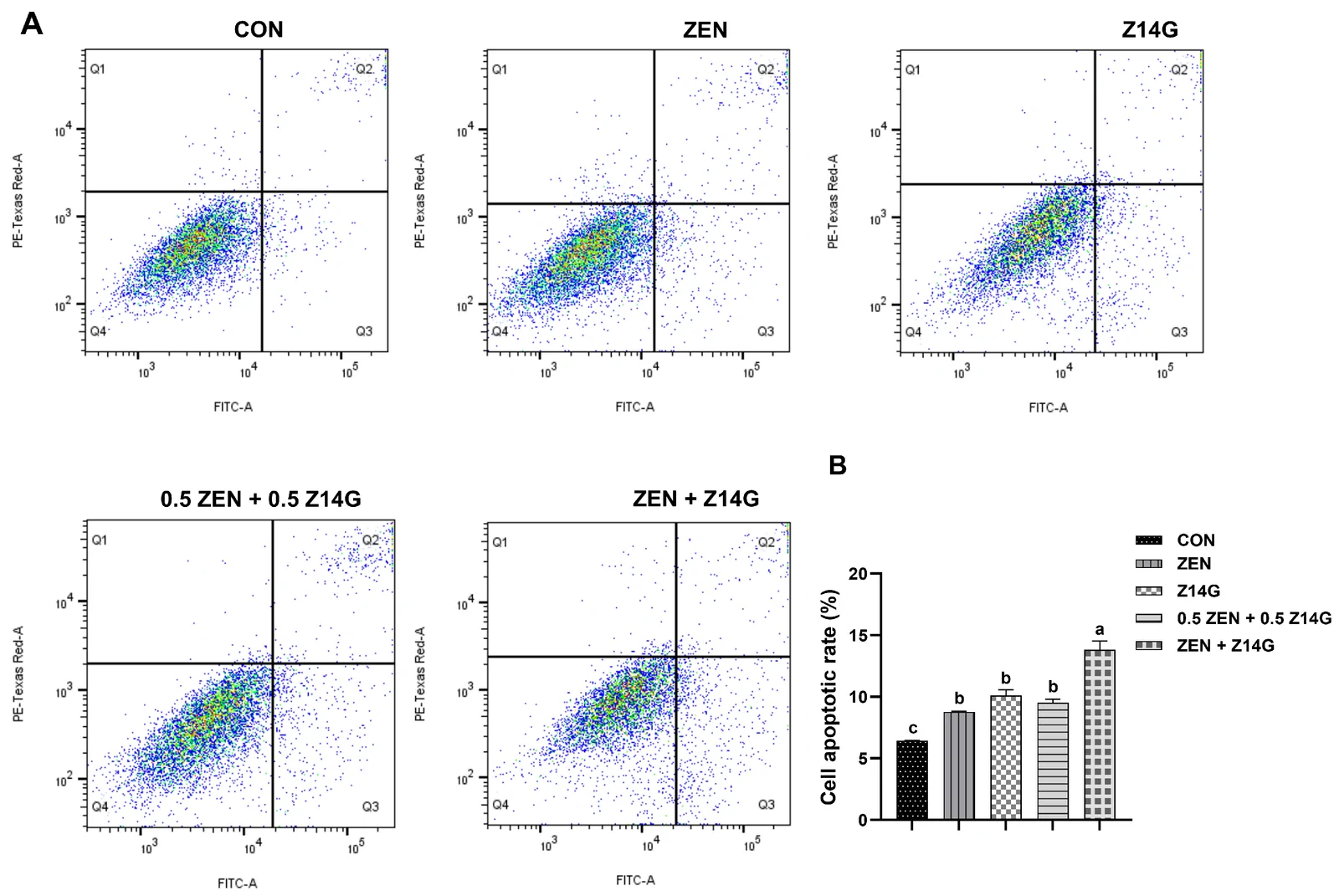
Effects of ZEN and Z14G exposure on apoptosis in IPEC-J2 cells. (**A**) represents the flow cytometry analysis of apoptosis in IPEC-J2 cells following individual and combined ZEN and Z14G exposure. Green fluorescence indicates Annexin V-FITC staining, while red fluorescence indicates PI staining. Q4 represents viable cells (Annexin V−/PI−), Q3 represents early apoptotic cells (Annexin V+/PI−), Q2 represents late apoptotic/secondary necrotic cells (Annexin V+/PI+), and Q1 represents necrotic cells (Annexin V−/PI+). (**B**) represents the quantification of total apoptotic cells, calculated as the sum of early apoptotic and late apoptotic/secondary necrotic cells shown in (**A**). *n* = 6. ^a–c^ Different lowercase letters indicate significant differences (*p* < 0.05).

The effects of ZEN and Z14G alone and in combination on the apoptotic cell population of IPEC-J2 cells are shown in Figure 2. Compared with the CON group, treatment with ZEN, Z14G, 0.5 ZEN + 0.5 Z14G, or ZEN + Z14G significantly increased the proportion of apoptotic cells (*p* < 0.05). The ZEN + Z14G co-exposure group showed the highest proportion of apoptotic cells, which was significantly higher than those observed in the other treatment groups (*p* < 0.05).

#### 3.1.3. The mRNA Expression of Cell Renewal-Related Genes

**Figure 3:**
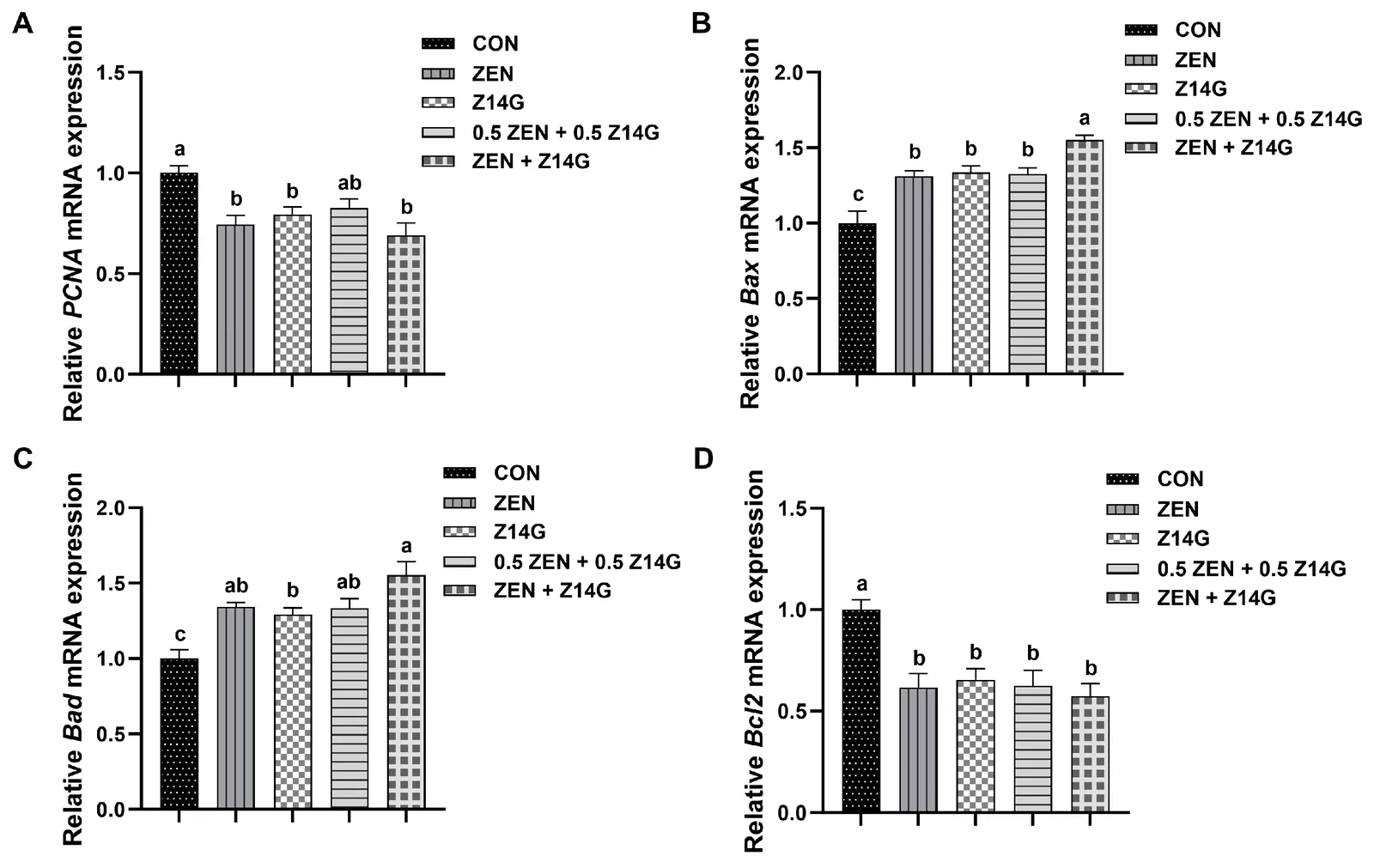
Effects of ZEN and Z14G exposure on cell renewal-related gene mRNA expression. (**A**–**D**) represent the mRNA expressions of *PCNA*, *Bax*, *Bad* and *Bcl2*, respectively. *n* = 6. ^a–c^ Different lowercase letters indicate significant differences (*p* < 0.05).

The effects of ZEN and Z14G alone and in combination on the mRNA expression of cell renewal-related genes in IPEC-J2 cells were shown in Figure 3. Compared with the CON group, treatment with ZEN, Z14G, or ZEN + Z14G significantly decreased the mRNA expression of *PCNA* and *Bcl2* while significantly increasing the mRNA expression of *Bax* and *Bad* in IPEC-J2 cells (*p* < 0.05). Most notably, these effects were most pronounced in the high-dose co-exposure group (*p* < 0.05). Treatment with 0.5 ZEN + 0.5 Z14G significantly decreased *Bcl2* mRNA expression while increasing *Bax* and *Bad* mRNA expression (*p* < 0.05), with no significant difference observed in *PCNA* mRNA expression (*p* > 0.05).

### 3.2. Cellular Injury and Barrier-Associated Responses to ZEN and Z14G in IPEC-J2 Cells

**Figure 4:**
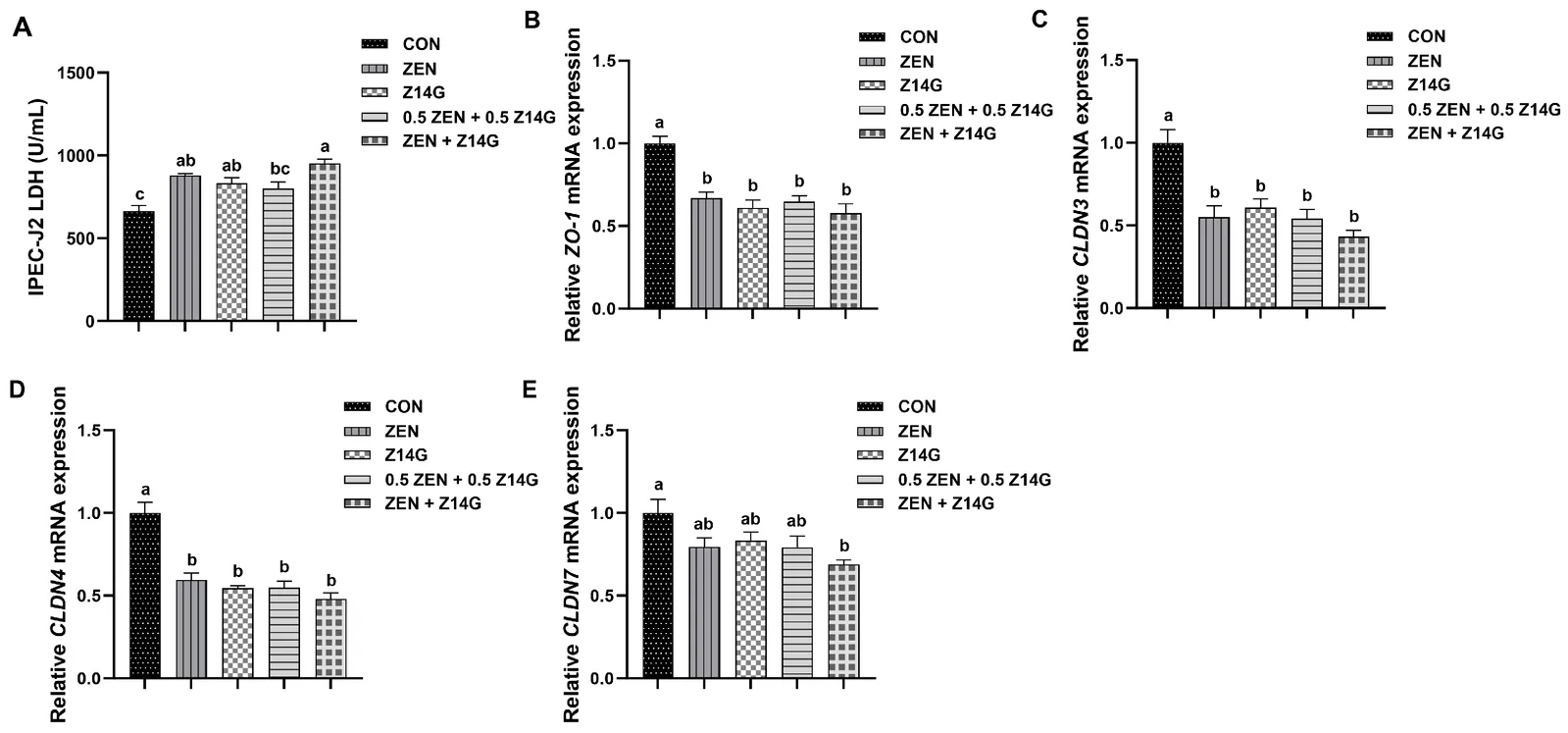
Effects of ZEN and Z14G exposure on LDH activity in the culture medium of IPEC-J2 cells and the mRNA expression levels of barrier-associated genes. (**A**) represents the detection of cell LDH activity. (**B**–**E**) represent the mRNA expression of ZO-1, CLDN3, CLDN4 and CLDN7, respectively. *n* = 6. ^a–c^ Different lowercase letters indicate significant differences (*p* < 0.05).

The effects of ZEN and Z14G exposure on LDH activity in IPEC-J2 cell culture medium and the mRNA expression of barrier-associated genes in IPEC-J2 cells are shown in Figure 4. The LDH activity in IPEC-J2 cell culture medium is shown in Figure 4A. Compared with the CON group, treatment with ZEN, Z14G, 0.5 ZEN + 0.5 Z14G, or ZEN + Z14G significantly increased the LDH activity of IPEC-J2 culture medium (*p* < 0.05). There were no significant differences among the mycotoxin groups (*p* > 0.05). The mRNA expression of barrier-associated genes in IPEC-J2 cells is shown in Figure 4B–E. Compared with the CON group, treatment with ZEN + Z14G significantly reduced mRNA expression levels of ZO-1, CLDN3, CLDN4, and CLDN7 in IPEC-J2 cells (*p* < 0.05). ZEN, Z14G, and 0.5 ZEN + 0.5 Z14G significantly reduced mRNA expression levels of ZO-1, CLDN3, and CLDN4 (*p* < 0.05), while no significant difference was observed in CLDN7 mRNA expression (*p* > 0.05).

The effects of ZEN and Z14G exposure on LDH activity in the culture medium and the mRNA expression of barrier-associated genes in IPEC-J2 cells are shown in Figure 4. The LDH activity in the culture medium is shown in Figure 4A. Compared with the CON group, treatment with ZEN, Z14G, 0.5 ZEN + 0.5 Z14G, or ZEN + Z14G significantly increased LDH activity in the culture medium (*p* < 0.05). No significant differences in LDH activity were observed among the mycotoxin-treated groups (*p* > 0.05). The mRNA expression levels of barrier-associated genes in IPEC-J2 cells are shown in Figure 4B–E. Compared with the CON group, treatment with ZEN + Z14G significantly reduced the mRNA expression levels of ZO-1, CLDN3, CLDN4, and CLDN7 in IPEC-J2 cells (*p* < 0.05). Treatment with ZEN, Z14G, or 0.5 ZEN + 0.5 Z14G significantly reduced the mRNA expression levels of ZO-1, CLDN3, and CLDN4 (*p* < 0.05), whereas no significant differences were observed in CLDN7 mRNA expression compared with the CON group (*p* > 0.05).

### 3.3. Oxidative Stress and Antioxidant Response of Individual and Combined Mycotoxins in IPEC-J2 Cells

#### 3.3.1. Intracellular Oxidative Stress and Antioxidant Capacity in IPEC-J2 Cells

**Figure 5:**
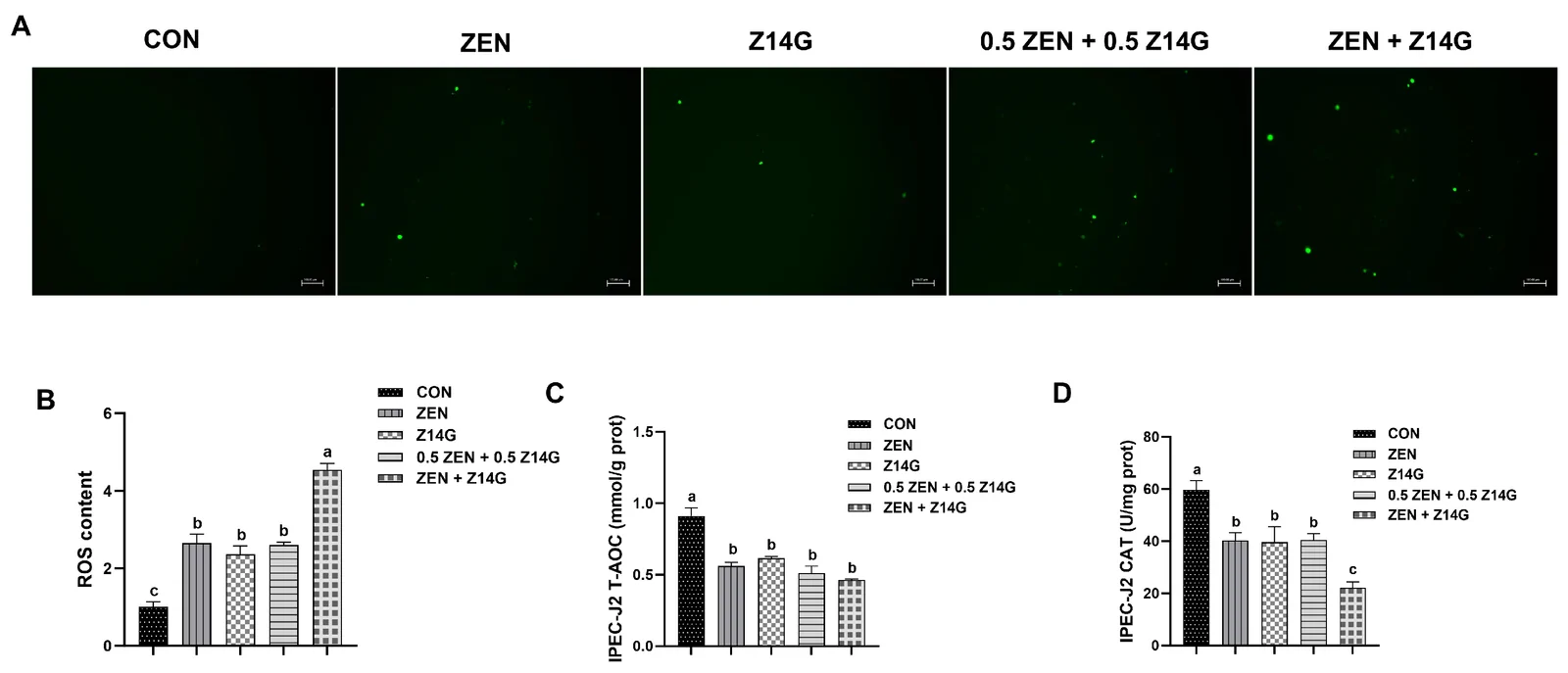
Effects of ZEN and Z14G exposure on intracellular oxidative stress and antioxidant capacity in IPEC-J2 cells. (**A**) represents fluorescence microscopy images of DCFH-DA fluorescence in IPEC-J2 cells. (**B**) represents the quantification of DCFH-DA fluorescence intensity shown in (**A**). (**C**) represents the total antioxidant capacity in IPEC-J2 cells. (**D**) represents catalase activity in IPEC-J2 cells. *n* = 6. ^a–c^ Different lowercase letters indicate significant differences (*p* < 0.05).

To assess oxidative stress following mycotoxin exposure, intracellular oxidative stress was evaluated using the DCFH-DA fluorescence assay in IPEC-J2 cells. The DCFH-DA fluorescence intensity in IPEC-J2 cells is shown in Figure 5A,B. Compared with the CON group, treatment with ZEN, Z14G, 0.5 ZEN + 0.5 Z14G, or ZEN + Z14G significantly increased DCFH-DA fluorescence intensity in IPEC-J2 cells (*p* < 0.05). Notably, the highest DCFH-DA fluorescence intensity was observed in the ZEN + Z14G co-exposure group. To further evaluate the effects of mycotoxin exposure on the cellular antioxidant defense system, CAT activity and T-AOC were measured in IPEC-J2 cells. As shown in Figure 5C,D, compared with the CON group, ZEN, Z14G, 0.5 ZEN + 0.5 Z14G, or ZEN + Z14G significantly decreased CAT activity and T-AOC in IPEC-J2 cells (*p* < 0.05). The ZEN + Z14G co-exposure group showed the most pronounced reduction in CAT activity.

#### 3.3.2. The mRNA Expression of Antioxidant-Related Genes

**Figure 6:**
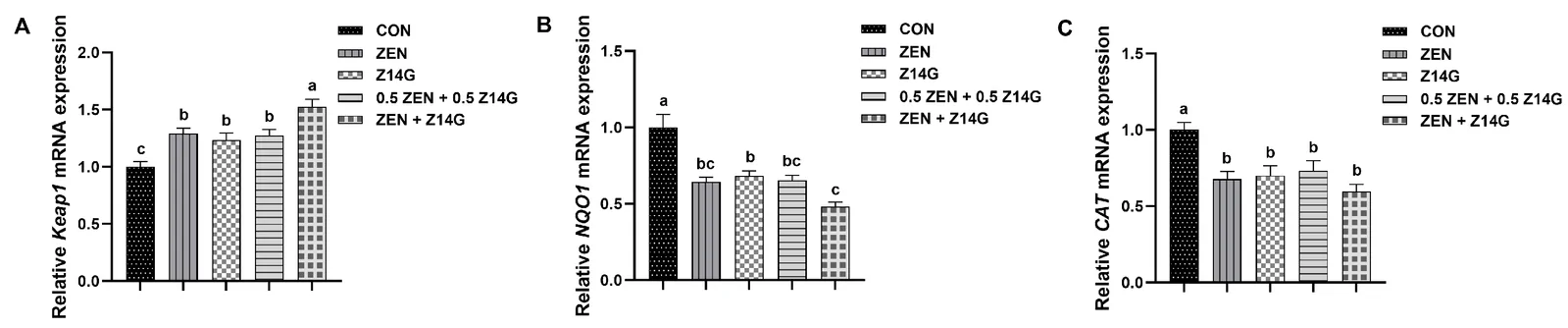
Effects of ZEN and Z14G exposure on the mRNA expression of antioxidant-related genes of IPEC-J2 cells. (**A**–**C**) represent the mRNA expressions of *Keap1*, *NQO1* and *CAT*, respectively. *n* = 6. ^a–c^ Different lowercase letters indicate significant differences (*p* < 0.05).

To further characterize the antioxidant defense response following mycotoxin exposure, we examined the mRNA expression of *Keap1*, *NQO1*, and *CAT*. Compared with the CON group, treatment with ZEN, Z14G, 0.5 ZEN + 0.5 Z14G, or ZEN + Z14G significantly increased the mRNA expression of *Keap1* and significantly decreased the mRNA expression of *NQO1* and *CAT* in IPEC-J2 cells (*p* < 0.05) (Figure 6). The ZEN + Z14G co-exposure group showed the most pronounced changes in *Keap1* and *NQO1* mRNA expression.

### 3.4. Inflammation-Related Gene Expression in IPEC-J2 Cells Following Individual and Combined Mycotoxin Exposure

**Figure 7:**
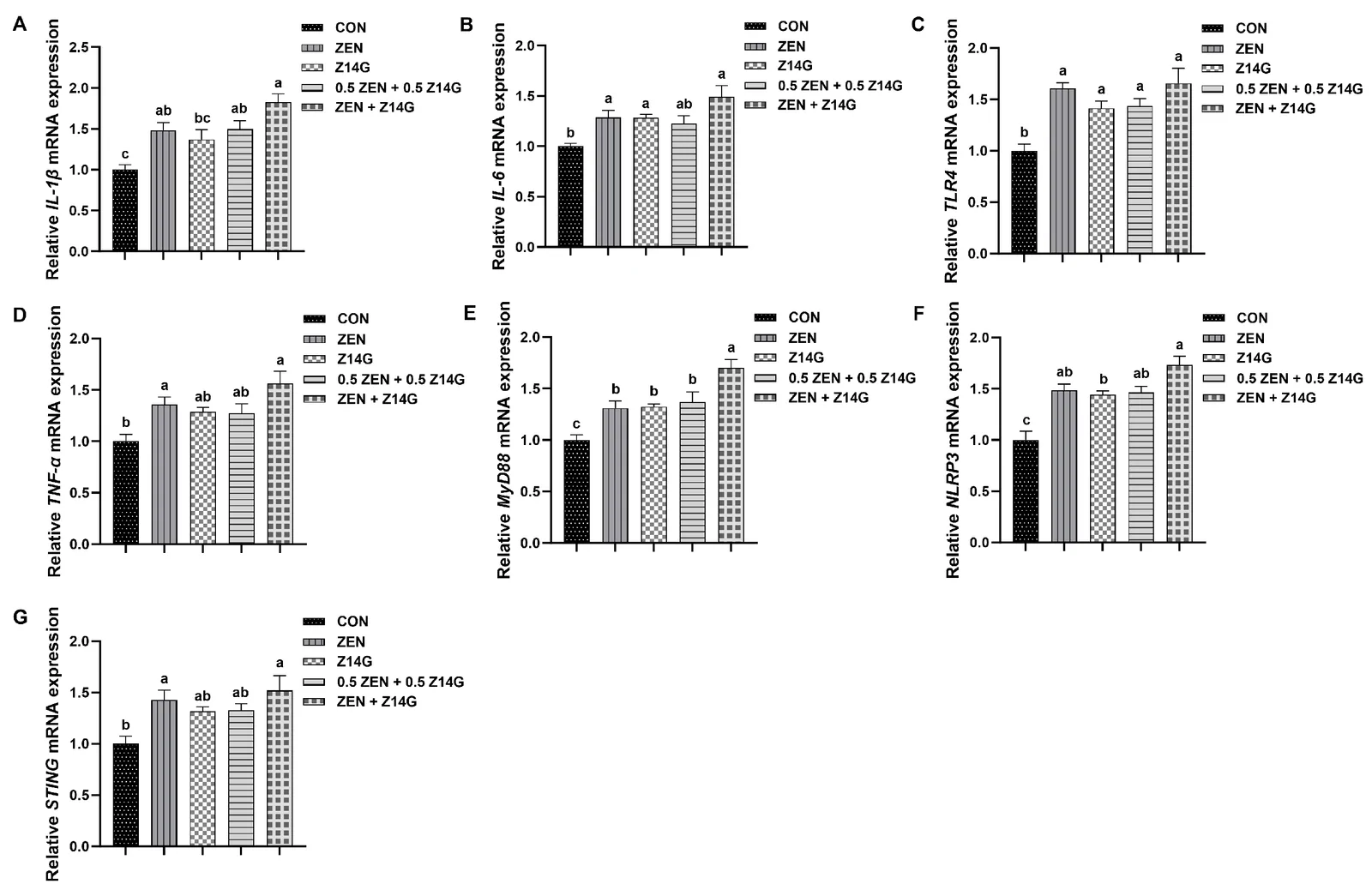
The effects of ZEN and Z14G exposure on the mRNA expression of inflammation-related genes of IPEC-J2 cells. (**A**–**G**) represent the mRNA expressions of *IL-1β*, *IL-6*, *TLR4*, *TNF-α*, *MyD88*, *NLRP3*, and *STING*, respectively. *n* = 6. ^a–c^ Different lowercase letters indicate significant differences (*p* < 0.05).

The effects of ZEN and Z14G exposure on the mRNA expression of inflammation-related genes in IPEC-J2 cells are shown in Figure 7. Compared with the CON group, both ZEN and ZEN + Z14G significantly increased the mRNA expression of *IL-1β*, *IL-6*, *TLR4*, *TNF-α*, *MyD88*, *NLRP3*, and *STING* in IPEC-J2 cells (*p* < 0.05). In addition, Z14G significantly increased the mRNA expression of *IL-6*, *TLR4*, *MyD88*, and *NLRP3* (*p* < 0.05), whereas no significant differences were observed in the mRNA expression of *IL-1β*, *TNF-α*, or *STING* (*p* > 0.05). Moreover, treatment with 0.5 ZEN + 0.5 Z14G significantly increased the mRNA expression of *IL-1β*, *TLR4*, *MyD88*, and *NLRP3* (*p* < 0.05), whereas no significant differences were observed in the mRNA expression of *IL-6*, *TNF-α*, or *STING* (*p* > 0.05). Overall, the ZEN + Z14G group showed the most pronounced changes in the expression of the inflammation-related genes examined.

## 4. Discussion

### 4.1. Effects of ZEN and Z14G on Cellular Homeostasis and Renewal in IPEC-J2 Cells

The underdeveloped gastrointestinal tracts of piglets are susceptible to damage from changes in diet and environment, thereby increasing the risk of disease [18]. To investigate the individual and combined toxic effects of ZEN and Z14G on the intestinal epithelium at the cellular level, this study utilized an IPEC-J2 cell model. Through dose-dependent screening of IPEC-J2 cells, we found that treatment with ZEN and Z14G resulted in a dose-dependent decrease in cell viability. Specifically, treatment with 80 μmol/L ZEN or 100 μmol/L Z14G for 24 h reduced cell viability by 36% and 30%, respectively. Based on these dose–response results, these concentrations were selected for subsequent experiments because they produced measurable and relatively comparable cytotoxic effects while retaining sufficient viable cells for downstream analyses. Importantly, these concentrations were selected as experimental concentrations for mechanistic investigation and should not be interpreted as equivalent biological doses or direct representations of physiological or dietary exposure levels. Differences in absorption, metabolism, intestinal transformation, and exposure between the in vitro culture system and the in vivo setting should therefore be considered when extrapolating the present findings to realistic exposure conditions. In the combined exposure assay, both half-dose and full-dose combination treatments significantly reduced cell viability. Concurrently, both single and combined exposures to ZEN and Z14G significantly inhibited the proliferation of IPEC-J2 cells and induced apoptosis, consistent with previous reports [13,19,20].

To maintain the integrity of the intestinal barrier, the epithelial cells undergo rapid turnover at a rate of once every 3–5 days, making them the fastest-renewing tissue in the body [21]. This characteristic also makes the intestine highly sensitive to toxins, providing an ideal starting point for studying how toxins interfere with cell regeneration. Studies have shown that ZEN exposure reduced PCNA expression in piglet intestinal villi and impaired small intestinal stem cell proliferative capacity [22]. Consistently, the present study revealed that, except for the low-dose combination, both individual and combined exposures to ZEN and Z14G significantly reduced *PCNA* mRNA expression in IPEC-J2 cells, suggesting that ZEN and Z14G inhibited IPEC-J2 cell proliferation. Abnormal apoptosis in IPEC-J2 cells not only aggravated intestinal tissue injury but also disrupted the intestinal barrier integrity. Thus, the precise regulation of apoptosis is critical for intestinal homeostasis, as established previously [23]. This process is centrally governed by the Bcl-2 family of proteins, which includes pro-apoptotic members such as Bad, Bax and Bid, and anti-apoptotic representatives like Bcl-2 and Bcl-XL [24]. Both individual and combined ZEN and Z14G exposure significantly altered the mRNA expression of the above apoptosis-related genes. This was consistent with previous reports showing that ZEN exposure increased *Bax* and decreased *Bcl-2* mRNA expression in PK15 cells [25]. Similarly, it was observed in zebrafish that ZEN induced excessive ROS accumulation, which led to DNA damage and oxidative stress by activating the p53 pathway and down-regulating Bcl-2 mRNA expression [26]. Collectively, consistent with the observed effects on proliferation and apoptosis, individual and combined exposure to ZEN and Z14G disrupts intestinal epithelial homeostasis by perturbing the critical balance between cell proliferation and apoptosis, thereby compromising epithelial renewal and overall intestinal stability.

### 4.2. Cellular Injury and Barrier-Associated Responses to ZEN and Z14G in IPEC-J2 Cells

The integrity of the intestinal epithelial barrier is essential for maintaining selective permeability and protecting against luminal insults [27,28]. LDH is a commonly used indicator of cellular injury and cytotoxicity [29,30]. In the present study, ZEN and Z14G exposure significantly increased LDH activity in the culture medium of IPEC-J2 cells, suggesting increased cellular injury following toxin exposure. Given the potential link between cellular injury and epithelial barrier dysfunction, we further examined the mRNA expression of several tight-junction-associated genes to assess the effects of ZEN and Z14G exposure on intestinal epithelial barrier function. The intestinal epithelial barrier is maintained by a complex network of structural proteins, including zonula occludens (ZOs), claudins (CLDNs), and occludin, which contribute to the regulation of epithelial integrity and selective permeability [31]. Among the claudin family members examined in the present study, CLDN3, CLDN4, and CLDN7 have been implicated in the maintenance of epithelial barrier properties and cellular homeostasis [32,33,34]. Previous studies have shown that ZEN exposure can downregulate the mRNA expression of several tight-junction-associated genes, including *ZO-1*, *CLDN1*, *OCLN*, *CLDN2*, *CLDN3*, and *CLDN4*, in the porcine intestine [35,36,37]. Consistent with these findings, we observed that ZEN and Z14G, either individually or in combination, significantly reduced the mRNA expression of *ZO-1*, *CLDN3*, and *CLDN4*. Notably, CLDN7 expression was significantly reduced only following combined exposure, suggesting that the combined exposure may have a greater effect on CLDN7 expression than either individual exposure. However, because only mRNA expression was assessed, these findings should be interpreted as alterations in barrier-associated gene expression rather than direct evidence of impaired epithelial barrier function.

### 4.3. Oxidative Stress and Antioxidant Response of Individual and Combined Mycotoxins in IPEC-J2 Cells

Oxidative stress has been implicated in the cellular responses to ZEN and Z14G exposure. Excessive oxidative stress can contribute to cellular injury by affecting membrane integrity, enzyme function, and other cellular processes [38]. Consistent with previous findings that 40 μmol/L ZEN induced oxidative stress in IPEC-J2 cells [17], we found that ZEN and Z14G, either individually or in combination, significantly increased DCFH-DA fluorescence intensity in IPEC-J2 cells. These findings suggest that both mycotoxins are associated with increased intracellular oxidative stress. However, DCFH-DA fluorescence is not specific to any particular ROS species and does not provide a direct quantitative measure of total intracellular ROS. Therefore, the increased DCFH-DA fluorescence observed in the present study should be interpreted as evidence of increased intracellular oxidative stress rather than a direct measurement of total ROS levels. To further characterize the cellular consequences of oxidative stress, we evaluated key indicators of antioxidant capacity. CAT activity and T-AOC levels are commonly used to reflect the cellular antioxidant status. In the present study, toxin exposure significantly reduced CAT activity and T-AOC levels in IPEC-J2 cells. This alteration in antioxidant capacity is consistent with previous reports showing that ZEN exposure decreased CAT, SOD, and GSH-Px activities and increased MDA content in the duodenum, jejunum, and IPEC-J2 cells [39,40,41].

We further examined the expression of genes associated with cellular antioxidant defense, including *Keap1*, *NQO1*, and *CAT*. The Keap1/Nrf2 axis is an important regulatory system involved in cellular antioxidant and detoxification responses [42]. NQO1 and CAT are among the antioxidant defense-related genes that contribute to the maintenance of redox homeostasis [43,44]. In the present study, both individual and combined exposure to ZEN and Z14G significantly increased *Keap1* mRNA expression while decreasing the mRNA expression of *NQO1* and *CAT* in IPEC-J2 cells. These changes indicate an alteration in the expression of genes associated with antioxidant defense following mycotoxin exposure. The observed pattern is consistent with previous reports of increased *Keap1* and decreased *CAT* expression following ZEN exposure in rabbit liver [27]. However, decreased *Keap1* and increased *NQO1* expression have also been reported in the duodenum of ZEN-exposed piglets [45]. Such differences may reflect tissue- and context-dependent regulation of antioxidant defense responses under different experimental conditions. Importantly, because only mRNA expression of selected antioxidant defense-related genes was assessed in the present study, these findings do not directly establish activation or inhibition of the Keap1/Nrf2 signaling pathway. Further studies assessing Nrf2 protein expression, nuclear translocation, and downstream target proteins would help clarify the involvement of this pathway in ZEN- and Z14G-induced oxidative stress.

### 4.4. Inflammation-Related Gene Expression in IPEC-J2 Cells Following Individual and Combined Mycotoxin Exposure

Oxidative stress in the intestine has been closely associated with inflammatory responses, and pattern recognition receptors such as TLR4 may participate in the regulation of inflammation. TLR4 signaling involves the adaptor protein MyD88 and can subsequently regulate downstream inflammatory responses, including the expression of pro-inflammatory cytokines [46]. In the present study, ZEN exposure significantly increased the mRNA expression of *TLR4*, *MyD88*, *IL-1β*, *TNF-α*, and IL-6, whereas Z14G increased the expression of *TLR4*, *MyD88*, and *IL-6* but did not significantly alter *TNF-α* or *IL-1β* expression. These findings are consistent with previous reports describing the pro-inflammatory effects of ZEN, including increased cytokine production in mice [47] and alterations in NF-κB-related inflammatory signaling [48]. The increased expression of pro-inflammatory cytokines may contribute to an inflammatory cellular environment and may be associated with alterations in epithelial barrier-related responses following mycotoxin exposure [49]. Notably, Z14G alone did not significantly alter the mRNA expression of *TNF-α* or *IL-1β*, suggesting that its inflammatory response profile may differ from that of ZEN.

In addition to the TLR4/MyD88-related response, the NLRP3 inflammasome is an important component of innate immune signaling and has been implicated in intestinal inflammation. Previous studies have reported that ZEN exposure can promote ROS accumulation and is associated with NLRP3 inflammasome activation and subsequent inflammatory responses [50]. In the present study, ZEN and Z14G exposure significantly increased *NLRP3* mRNA expression, suggesting an association between mycotoxin exposure and NLRP3-related inflammatory signaling. However, because only *NLRP3* mRNA expression was measured, the present findings do not directly demonstrate NLRP3 inflammasome activation or Caspase-1-mediated cytokine maturation. Similarly, STING is an important cytosolic DNA-sensing component involved in innate immune regulation, and its dysregulation has been associated with inflammatory responses [51]. Previous studies have reported that ZEN exposure can increase STING expression and affect NF-κB-related inflammatory responses [52,53,54]. Consistent with these observations, ZEN and full-dose ZEN + Z14G exposure significantly increased STING mRNA expression in IPEC-J2 cells, whereas no significant changes were observed following Z14G alone or half-dose co-exposure. These findings suggest that the expression of STING may be differentially responsive to the dose and composition of mycotoxin exposure. Overall, the observed changes in *TLR4*, *MyD88*, *NLRP3*, and *STING* expression support the involvement of innate immune and inflammatory signaling responses following ZEN and Z14G exposure, although the specific contribution and causal relationships of these pathways require further investigation.

Several limitations should be acknowledged. First, the present study was conducted exclusively using the IPEC-J2 cell model, which provides a useful system for investigating direct effects on porcine intestinal epithelial cells but does not fully recapitulate the complexity of the in vivo intestinal environment, including intestinal metabolism, microbiota interactions, and systemic immune responses. Second, the stability and potential deglucosylation of Z14G to ZEN were not directly assessed in the present culture system; therefore, the contribution of Z14G conversion to the observed effects cannot be completely excluded. Third, the pronounced cytotoxicity observed following co-exposure may have contributed to some of the downstream molecular changes, and the single exposure time point used in the present study does not establish temporal or causal relationships among oxidative stress, inflammatory responses, and barrier-associated alterations. Finally, the present study relied primarily on transcriptional and biochemical measurements, and therefore did not comprehensively validate protein-level changes or pathway-specific mechanisms. Further studies incorporating appropriate in vivo models, assessment of Z14G stability and metabolism, time-course experiments, and protein-level or pathway-specific functional analyses are warranted. Moreover, the concentrations used in the present in vitro study were selected based on cellular dose–response characteristics and were not intended to directly reproduce dietary exposure levels. Therefore, the present findings should not be directly extrapolated to in vivo toxicity or practical dietary risk without considering gastrointestinal dilution, absorption, metabolism, and other exposure-related factors.

## 5. Conclusions

In conclusion, Z14G exhibited weaker cytotoxicity than ZEN when administered individually under the conditions tested in IPEC-J2 cells, whereas co-exposure to ZEN and Z14G resulted in more pronounced cytotoxicity than either individual exposure, with ZEN appearing to contribute substantially to the observed response. Both individual and combined exposures were associated with increased oxidative stress, altered antioxidant responses, and changes in inflammation- and barrier-associated gene expression. These findings suggest an interconnected cellular response involving oxidative stress, inflammatory responses, and alterations in intestinal epithelial barrier-associated responses. Overall, this study highlights the importance of considering the combined effects of ZEN and its masked forms in risk assessment and provides evidence that ZEN may contribute substantially to the enhanced effects observed during co-exposure.

## Figures and Tables

**Table 1:** List of primers used in quantitative real-time PCR.

Gene	Primer Sequence (5′ to 3′)	Accession No.	Size, bp
*PCNA*	F: GAGGAGGAAGCAGTTACCATAGA	NM_001291925.1	119
	R: GACATACTGAGTGTGACTGTAGGA		
*Bax*	F: ATGATCGCAGCCGTGGACACG	XM_013998624.2	296
	R: ACGAAGATGGTCACCGTCTGC		
*Bad*	F: GAGTCGCCACAGCTCTTACC	XM_021082883.1	188
	R: GGCGAGGAAGTCCCTTCTTGA		
*Bcl2*	F: CTTCAGGGATGGGGTGAACT	XM_021099593.1	200
	R: GCCCATACAGCTCCACAAAG		
*Keap1*	F:GTGTTACTACCCAGAGAGGAATGA	NM_001114671.1	104
	R: CCGCAGCATAGATACAGTTGTG		
*NQO1*	F: GCAGAGAGTACATGGAGCCA	NM 001159613.1	131
	F:ATCAGGTCAGCCGCTTCAATC		
*CAT*	F: AGATGGACACAGGCACATGA	NM_214301.2	172
	R: CCGGATGCCATAGTCAGGAT		
*IL-1β*	F: TTGTCTGTGATGCCAACGTG	XM_021085847.1	108
	R: TGAGGAGGTGGAGAGCCTTC		
*IL-6*	F: ACCGGTCTTGTGGAGTTTCA	NM_001252429.1	170
	R: GCATTTGTGGTGGGGTTAGG		
*TLR4*	F: CTCCAGCTTTCCAGAACTGC	NM_001113039.2	192
	R: AGGTTTGTCTCAACGGCAAC		
*TNF-α*	F: CCAATGGCAGAGTGGGTATG	JF831365.1	116
	R: TGAAGAGGACCTGGGAGTAG		
*MyD88*	F: ATTGAAAAGAGGTGCCGTCG	NM_001099923.1	188
	R: CAGACAGTGATGAACCGCAG		
*NLRP3*	F: GTTGCATATTCTCAGCGATTGG	NM_001256770.2	122
	R: TCTCCCAGGCTGTTACCACT		
*STING*	F: AGCAGATAGGACTGCTGGTG	NM_001142838.1	78
	R: TGCTCTGGTACCTGGAGTG		
*ZO-1*	F: GCATGATGATCGTCTGTCCTACC	XM_013993251.1	108
	R: CCGCCTTCTGTATCTGTGTCTTC		
*CLDN3*	F: CTACGACCGCAAGGACTACG	NM_001160075.1	120
	R: TAGCATCTGGGTGGACTGGT		
*CLDN4*	F: CGCCCTCATCGTCATCTGTA	NM_001161637.1	94
	R: GCTCTCATCATCCACGCAGT		
*CLDN7*	F: TTTCATCGTGGCAGGTCTTTG	NM_001160076.1	138
	R: CCTGCCCAGCCAATGAAGAT		
*β-Actin*	F: GGACTTCGAGCAGGAGATGG	NM_001170517.2	138
	R: AGGAAGGAGGGCTGGAAGAG		

## Data Availability

The original contributions presented in this study are included in the article. Further inquiries can be directed to the corresponding author.

## Abbreviations

The following abbreviations are used in this manuscript:

ZEN	Zearalenone
Z14G	Zearalenone-14-glucoside
IPEC-J2	Intestinal porcine epithelial cell-jejunal 2
LDH	Lactate dehydrogenase
Bad	BCL-2-associated death
Bax	BCL-2-associated X
Bcl2	B-cell lymphoma protein 2
PCNA	Proliferating cell nuclear antigen
CLDN	Claudin
ZO-1	Zonula occludens-1
ROS	Reactive oxygen species
T-AOC	Total antioxidant capacity
CAT	Catalase
Keap1	Kelch-like ECH-associated protein
NQO1	NAD (P) H: quinone oxidoreductase 1
IL-1β	Interleukin-1β
IL-6	Interleukin-6
TLR4	Toll-like receptor 4
TNF-α	Tumor necrosis factor-α
MyD88	Myeloid differentiation factor 88
NLRP3	NLR family pyrin domain containing 3
STING	Stimulator of interferon genes
